# Characterization of non-heme iron aliphatic halogenase WelO5* from *Hapalosiphon welwitschii* IC-52-3: Identification of a minimal protein sequence motif that confers enzymatic chlorination specificity in the biosynthesis of welwitindolelinones

**DOI:** 10.3762/bjoc.13.115

**Published:** 2017-06-16

**Authors:** Qin Zhu, Xinyu Liu

**Affiliations:** 1 Department of Chemistry, University of Pittsburgh, 219 Parkman Avenue, Pittsburgh, PA 15260, USA

**Keywords:** alkaloid biogenesis, biosynthetic divergency, C–H activation, halogenase, non-heme iron enzyme

## Abstract

The in vitro biochemical characterization revealed that iron/2-oxoglutarate (Fe/2OG)-dependent aliphatic halogenase WelO5* in *Hapalosiphon welwitschii* IC-52-3 has an enhanced substrate specificity towards 12-*epi*-hapalindole C (**1**) in comparison to WelO5 in *H. welwitschii* UTEX B1830. This allowed us to define the origin of the varied chlorinated versus dechlorinated alkaloid structural diversity between the two welwitindolinone producers. Furthermore, this study, along with the recent characterization of the AmbO5 protein, collectively confirmed the presence of a signature sequence motif in the C-terminus of this newly discovered halogenase enzyme family that confers substrate promiscuity and specificity. These observations may guide the rational engineering and evolution of these proteins for biocatalyst application.

## Introduction

Carbon–halogen (C–X) bonds are prevalent structural motifs in modern agrochemicals, pharmaceuticals and bioactive natural products [1–2] and chlorination is the most common functionalization of this type [1–2]. Among other effects, chlorination enhances the electrophilicity of the modified carbon and alters the biological activities of drug(-like) molecules [3–4]. As aliphatic C–H groups are ubiquitous in organic molecules, synthetic transformations that allow for the selective modification of this type of functional group have been long sought after. While numerous methods have emerged that permit the late-stage functionalizations of inert aliphatic carbons with oxygen-containing functionalities [5–8], analogous oxidative functionalizations with halogens via C–H activations remain challenging that need to be addressed [9–11]. Recently, during the systematic investigation of hapalindole-type alkaloid biogenesis [12–19], we discovered a family of Fe/2OG-dependent halogenases that can oxidatively monochlorinate aliphatic carbon centers in freestanding molecules, typified by WelO5 protein in the biogenesis of welwitindolinones [17]. This discovery provided a new opportunity to utilize protein catalysts for late-stage halogenations of unactivated carbons in bioactive small molecules.

Although the initially characterized WelO5 has a restricted substrate scope [17], we have recently shown its homolog, AmbO5, in the biogenesis of ambiguines is capable of modifying seven structurally distinct hapalindole-type alkaloids [18]. The biochemical characterizations of WelO5/AmbO5 chimera revealed that a C-terminal sequence motif plays a role in the substrate tolerance and provided insights into the origin of substrate promiscuity in this family of proteins [18]. In this work, we report the characterization of the third WelO5-type protein, WelO5*, for the biogenesis of welwitindolinones in *H. welwitschii* IC-52-3. We show that WelO5* exhibits an enhanced specificity towards 12-*epi*-hapalindole C (**1**), a substrate poorly processed by WelO5 from *H. welwitschii* UTEX B1830, while maintaining its fidelity towards 12-*epi*-fischerindole U (**2**) as WelO5. This observation provided a molecular basis for the altered structural diversity of hapalindole-type alkaloids between the two welwitindolinone producers. The extreme sequence similarity (95% identical) between WelO5* and WelO5 allowed us to trace the origin of this observed specificity difference to 11 amino acid residues at a C-terminal sequence motif, initially discovered in the comparative characterization of WelO5 and AmbO5 [18]. This further confirms the functional significance of this conserved sequence motif in this new halogenase family that may guide the rational engineering and evolution of these proteins for biocatalyst application.

## Results and Discussion

**Figure 1:**
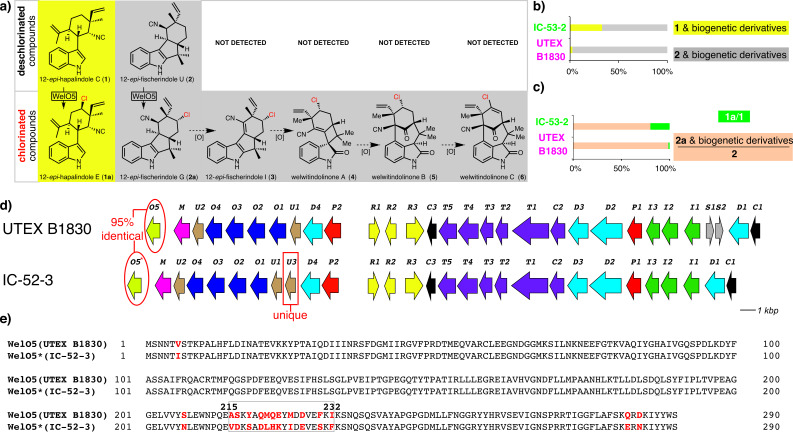
Comparative analysis of hapalindole-type alkaloids and their BGCs in two welwitindolinone producers implicates the functional role of Fe/2OG-dependent WelO5* halogenase in structural diversifications in *H. welwitschii* IC-52-3. a) Representative hapalindole-type alkaloids tabulated based on oxidation states in the welwitindolinone producers *H. welwitschii* UTEX B1830 and IC-52-3. b) Relative quantities of **1** and its biogenetic derivative **1a** versus **2** and its biogenetic derivatives (**2a**, **3**–**6**) in *H. welwitschii* IC-52-3 and UTEX B1830. c) Comparison of the ratio of chlorinated versus dechlorinated hapalindole-type alkaloids based on their biosynthetic origins (i.e., **1** or **2**) in *H. welwitschii* IC-52-3 and UTEX B1830. d) Comparison of welwitindolinone BGCs in *H. welwitschii* IC-52-3 and UTEX B1830. The tailoring enzyme coding genes, including *wel*U1-3, *wel*O1-5 and *wel*M, encoded in the welwitindolinone BGC from *H. welwitschii* IC-52-3, were renamed due to their extreme similarities to those from *H. welwitschii* UTEX B1830. e) Sequence comparison of WelO5 and WelO5*. Varied residues are highlighted in red with the most aggregated region 215-232 in grey rectangle.

*H. welwitschii* IC-52-3 and UTEX B1830 are two known welwitindolinone producers that were reported to produce identical sets of hapalindole-type alkaloids [20], albeit the detailed metabolite analysis from the latter was never published. During our recent effort to define the genetic and molecular basis of welwitindolinone biogenesis, we re-validated the profiles of hapalindole-type alkaloids in *H. welwitschii* UTEX B1830 as originally claimed [13]. This effort in turn led us to recognize there are two distinct differences in terms of hapalindole-type alkaloid structural diversities in these welwitindolinone producers. When the alkaloid molecules are grouped based on their biogenetic relatedness across two producing organisms (Figure 1a and Figure S1, Supporting Information File 1), the relative quantities of 12-*epi*-hapalindole C (**1**) and its biogenetic derivatives (i.e., 12-*epi*-hapalindole E (**1a**)) constitute more than 1/3 of the total hapalindole-type alkaloids isolated from *H. welwitschii* IC-52-3. This ratio is noticeably higher than what was observed for *H. welwitschii* UTEX B1830 (Figure 1b). Moreover, the ratio of chlorinated **1a** to dechlorinated **1** present in *H. welwitschii* IC-52-3 is significantly higher than that in *H. welwitschii* UTEX B1830 (Figure 1c). To understand the genetic and molecular basis for these differences in structural diversity, we compared the corresponding welwitindolinone biosynthetic gene clusters (BGCs) identified from *H. welwitschii* IC-52-3 and UTEX B1830 (Figure 1d) [13,21]. While the majority of the biosynthetic enzymes encoded in the two pathways are sequence-identical (Figure S2, Supporting Information File 1), two noticeable differences were identified (Figure 1d, red rectangle and circle highlights). First, the welwitindolinone pathway in *H. welwitschii* IC-52-3 contains a unique protein coded by *wel*U3 gene. We have recently characterized WelU3 protein by in vitro reconstitution and demonstrated it is a dedicated enzyme for the biosynthesis of **1** from 3-geranyl 3-(isocyanovinyl)indolenine [16], a common intermediate used in the biogenesis of all hapalindole-type alkaloids [15]. The presence of WelU3 in *H. welwitschii* IC-52-3 thus accounted for the observed increased quantities of **1** in this producer.

The second key difference between the two welwitindolinone BGCs resides on the halogenase coding gene *wel*O5. From the BLAST-P analysis, the protein sequence of WelO5 in *H. welwitschii* IC-52-3 is nearly (95%) identical to that in *H. welwitschii* UTEX B1830 (Figure 1d). Due to their close resemblance, we rename WelO5 in *H. welwitschii* IC-52-3 as WelO5* to facilitate the remaining discussion. Upon a close inspection of the sequence differences between WelO5* and WelO5, we realized that 11 out of the 15 varied amino acids fall into residues 215-232 (Figure 1e). We have shown the same type of C-terminal sequence motif in AmbO5, the Fe/2OG-dependent halogenase involved in the biogenesis of ambiguines, plays a role in its expanded substrate scope [18]. Moreover, our recent structural characterizations of WelO5 in the absence and presence of **2** have shown residues 215-232 of WelO5 encode an α-helical motif that helps keeping the small molecular substrate in the active site by undergoing a dramatic conformational change upon substrate binding [22]. Based on these earlier observations on the functional relevance of this C-terminal sequence motif in WelO5 and AmbO5 halogenases and the fact that the sequence of WelO5* differs near exclusively from that of WelO5 in the same region, we hypothesize that WelO5* must have an altered substrate preference to **1** and **2** to account for the observation that the ratio of chlorinated **1a** versus dechlorinated **1** present in *H. welwitschii* IC-52-3 is significantly higher than that in *H. welwitschii* UTEX B1830.

**Figure 2:**
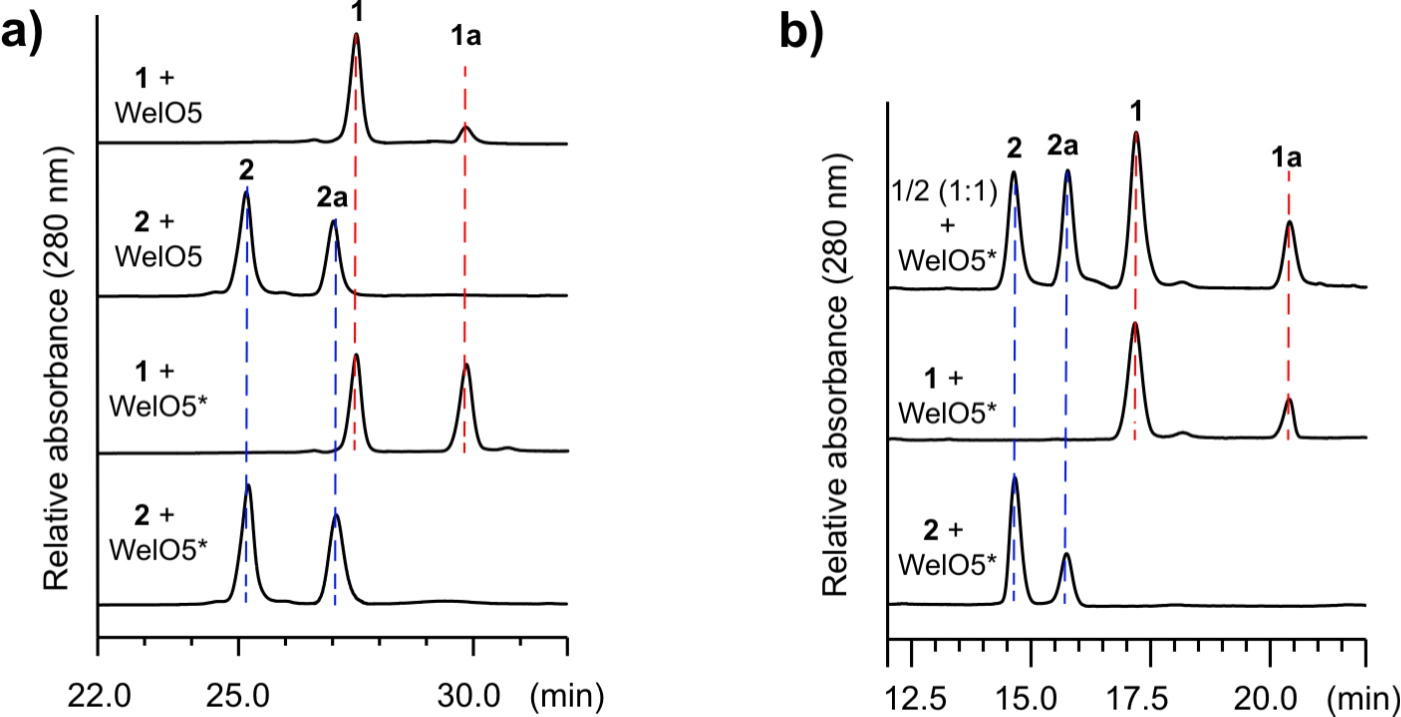
a) In vitro characterizations show that WelO5* has comparable activity to both **1** and **2**, distinct from WelO5. b) In vitro characterization of WelO5* substrate preference towards **1** and **2** using a substrate competition assay. For the HPLC data shown in a), assays were conducted with WelO5* or WelO5 (20 µM) with 0.5 mM of **1** or **2** for 20 min at 30 °C. HPLC was run with a C18 Luna column (Phenomenex 250 × 4.6 mm) with a solvent gradient from 60–90% methanol/water in 30 min at a flow rate 1 mL/min. For the HPLC data shown in b), assays were conducted with WelO5* (20 µM) with 0.5 mM of **1** and/or **2** for 10 min at 30 °C. HPLC was run with a XC-C18 Kinetex column (Phenomenex 150 × 2.6 mm) with an isocratic solvent 50% acetonitrile/water in 30 min at a flow rate 0.4 mL/min. These conditions were chosen to achieve a better separation of **1**/**1a**/**2**/**2a**.

To test this hypothesis, the *wel*O5* gene was synthesized and ligated into the expression vector pQTEV. Heterologous expression in *E. coli* and purification by immobilized metal affinity chromatography (IMAC) gave the N-terminal hepta-His-tagged WelO5* in a comparable yield (20 mg/L) as for WelO5 [11]. With abundant WelO5* in hand, we proceeded on its in vitro characterization using the assay conditions established for WelO5 and AmbO5 [18]. For a 100 µL scale reaction, WelO5* (20 µM final concentration) rapidly converted circa 50% of **1** and **2** (0.5 mM final concentration) to their chlorinated derivatives **1a** and **2a** within 20 min in the presence of cosubstrate 2OG, cofactor Fe(II) and molecular oxygen (Figure 2a, bottom two lanes). Under identical conditions, WelO5 showed a comparable conversion rate of **2** to **2a** but was much more sluggish towards **1** (Figure 2a, top two lanes), consistent with our previous observation [17]. While a full steady state kinetic analysis remains challenging due to the limited substrate availability, we assessed the *k*_obs_ of WelO5* towards **1** and **2**, as described for WelO5 and AmbO5 [18]. WelO5* exhibits nearly identical *k*_obs_ towards **1** (1.8 ± 0.1 min^−1^) and **2** (1.9 ± 0.2 min^−1^), distinct from WelO5 which prefers **2** (*k*_obs_ = 1.8 ± 0.2 min^−1^) to **1** (*k*_obs_ = 0.73 ± 0.08 min^−1^) [17]. These analyses collectively confirm WelO5* does have an enhanced substrate specificity towards **1** in comparison to WelO5, while maintaining its activity towards **2**.

To gain further insights into the substrate preference of WelO5*, complementary to the *k*_obs_ measurement, we assessed its in vitro activity towards an equimolar amount of **1** and **2** (Figure 2b). For a 100 µL scale reaction with an equal amount of **1** and **2** (0.25 mM final concentration for each molecule), WelO5* (20 µM final concentration) was able to convert ca. 50% of **2** to **2a** and 33% of **1** to **1a** within 10 min (Figure 2b), indicating that WelO5* has a higher affinity (ca. two-fold) towards **2** than **1** under the enlisted in vitro assay conditions. This observation augments the altered substrate specificity of WelO5* versus WelO5 as the molecular basis for the varied structural diversities observed between the two welwitindolinone producers (Figure 1c).

**Figure 3:**
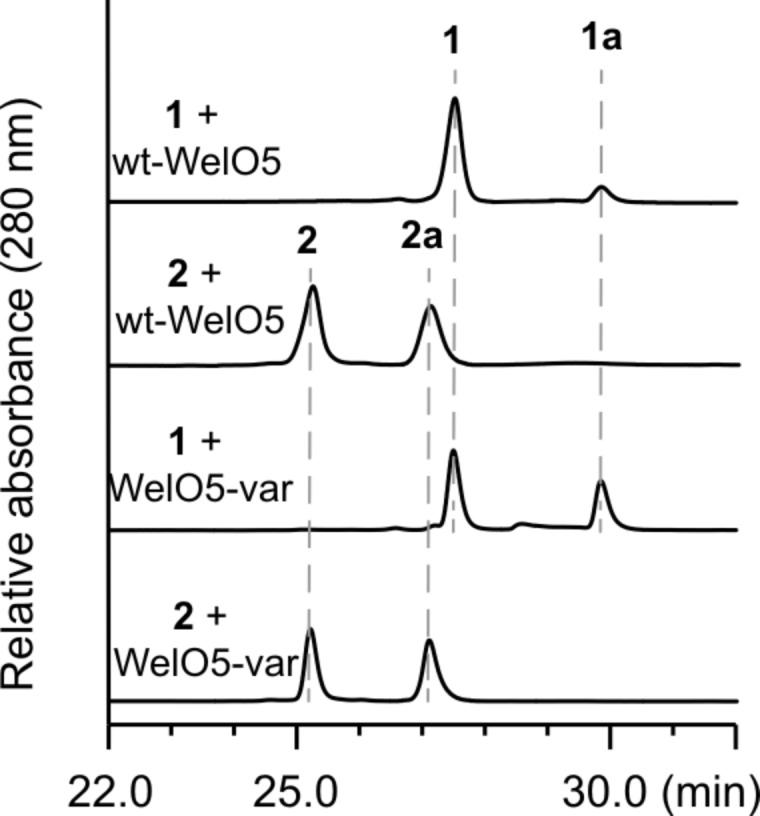
In vitro characterization of a WelO5 variant with enhanced specificity towards **1**. All of the HPLC data shown are in vitro assays conducted with WelO5* or its variants (20 µM) with 0.5 mM of **1** or **2** for 20 min at 30 °C. HPLC was run with a C18 Luna column (Phenomenex 250 × 4.6 mm) with a solvent gradient from 60–90% methanol/water in 30 min at a flow rate 1 mL/min.

As 11 out of the 15 amino acid variations between WelO5* and WelO5 fall into residues 215–232, a sequence motif that was shown previously to play a role in the expanded substrate scope of AmbO5 [7], we hypothesize the same motif in WelO5* may be important for its enhanced specificity towards **1**. To examine this hypothesis, we generated a variant of WelO5 (WelO5-*var*) by swapping its residues 215–232 to those in WelO5*. This variant was heterologously expressed and purified in an identical manner as the wild-type WelO5 and WelO5*. WelO5-*var* displayed a noticeably enhanced activity towards **1** based on the standard HPLC-based in vitro assay (Figure 3). The *k*_obs_ measurement (1.8 ± 0.2 min^−1^ for **1** and 1.8 ± 0.1 min^−1^ for **2**) indicates the activity of WelO5-*var* towards **1** is elevated to a comparable level as the wild type WelO5* while retaining its fidelity towards **2**. This result provides evidence that WelO5 can be rendered as specific as WelO5* towards **1** by replacing 11 varied amino acids between residues 215–232 with those of WelO5* and validates our hypothesis that the sequence variations between WelO5* and WelO5 at residues 215–232 are directly responsible for their enhanced or diminished specificity towards **1**.

## Conclusion

In summary, intrigued by the hapalindole-type alkaloid structural diversity difference between *H. welwitschii* IC-52-3 and UTEX B1830, we examined the enzymatic activity of Fe/2OG-dependent WelO5* halogenase. Although WelO5* is nearly sequence-identical to the previously characterized WelO5, it showed enhanced chlorination activity towards **1**, distinct from WelO5. This study, along with the recent characterizations of WelU1 and WelU3 enzymes in *H. welwitschii* IC-52-3 [16], collectively provides the molecular basis for the altered structural diversity between the two welwitindolinione producers. Furthermore, the close sequence similarity between WelO5* and WelO5 allowed us to reveal a C-terminal sequence motif (residues 215–232) that harbors 11 varied amino acids between the two proteins plays the most critical role on the observed enhanced activity of WelO5* towards **1**. While the mechanism underlying how this sequence motif controls the substrate tolerance and specificity, as seen in AmbO5 previously and WelO5* in this study, is a subject for future studies. However its presence in this newly discovered halogenase family provides an entry point for the rational engineering of these enzymes for tailoring small molecules beyond hapalindole-type alkaloids.

## Experimental

**Protein expression and purification:** Genes coding WelO5*, WelO5-*var* proteins were synthesized and ligated into pQTEV vector by BioBasic Inc. Heterologous expressions of WelO5* and WelO5-*var* in *E. coli* and subsequent purifications by IMAC were conducted in an identical fashion as described for WelO5 [17]. Protein homogeneities were assessed by SDS-PAGE analysis (Figure S3, Supporting Information File 1). The approximate yield for each protein is 20 mg/L.

**In vitro assay:** Substrates **1** and **2** were procured by isolation as previously described [17]. In vitro assays with a single or double small molecular substrate(s) (**1** and/or **2**) were conducted on a 100 µL scale, with 20 µM of the enzyme, 0.5 mM of the small molecular substrate(s) and appropriate cosubstrate/cofactors as described exactly for WelO5 and AmbO5 [18]. Reactions were stopped at 2 min (for *k*_obs_ estimation), 10 min (for Figure 2b) or 20 min (for Figure 2a/3) and extracted with EtOAc before subjecting to HPLC analysis as previously described [18]. Analytical reversed-phase HPLC was performed using a Dionex UHPLC with a photodiode array UV–vis detector (Thermo Fisher Scientific) using either a C18 Luna column (Phenomenex 250 × 4.6 mm) or a XC-C18 Kinetex column (Phenomenex 150 × 2.6 mm). The conversion ratio for each enzymatic assay from **1** to **1a** or **2** to **2a** was quantified by comparing the corresponding HPLC peak areas for compounds **1**, **1a**, **2** or **2a**, assuming their extinction coefficients at 280 nm are identical.

## Supporting Information

File 1 Additional figures.
