# Supplementary material for: Characterization of non-heme iron aliphatic halogenase WelO5* from Hapalosiphon welwitschii IC-52-3: Identification of a minimal protein sequence motif that confers enzymatic chlorination specificity in the biosynthesis of welwitindolelinones
Source: Beilstein J Org Chem. 2017 Jun 16;13:1168–73. doi: 10.3762/bjoc.13.115 (PMC5480362; doi:10.3762/bjoc.13.115)
Supplement: File 1 — Additional figures. [file Beilstein_J_Org_Chem-13-1168-s001.pdf]

Supporting Information

for

Characterization of non-heme iron aliphatic halogenase WelO5* from *Hapalosiphon welwitschii* IC-52-3: Identification of a minimal protein sequence motif that confers enzymatic chlorination specificity in the biosynthesis of welwitindolelinones

Qin Zhu and Xinyu Liu*

Address: Department of Chemistry, University of Pittsburgh, 219 Parkman Avenue, Pittsburgh, PA 15260, USA

Email: Xinyu Liu - xinyuliu@pitt.edu

* Corresponding author

Additional figures

a)

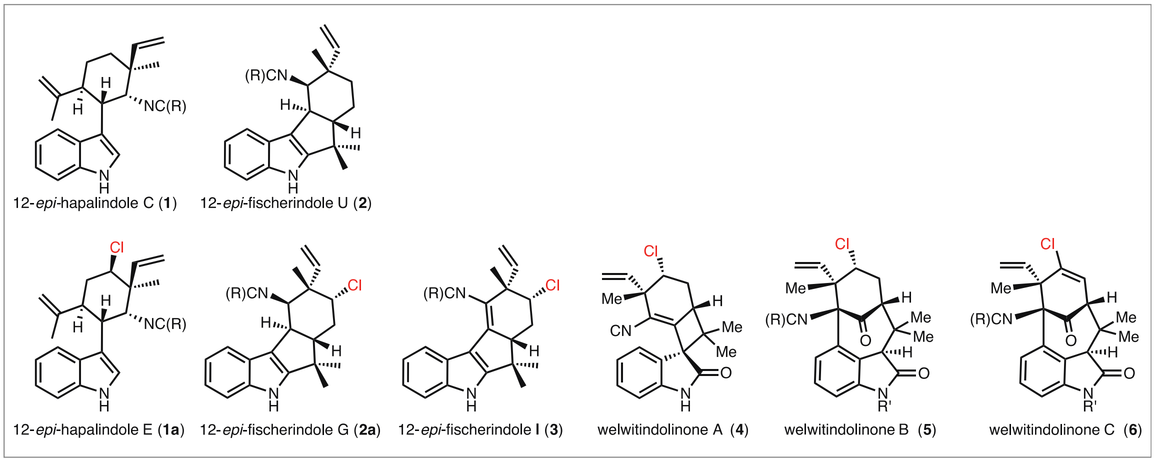

b)

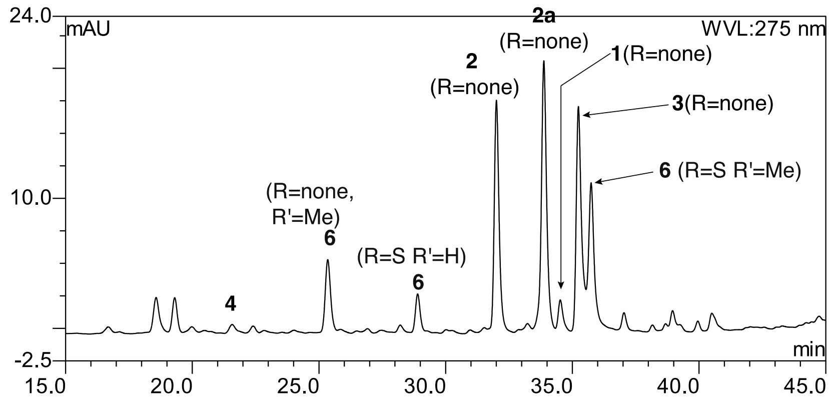

Compounds	%
**4** (R=none)	1.1
**6** (R=S, R'=H)	7.1
**6** (R=none, R'=Me)	3.3
**2** (R=none)	20.7
**2a** (R=none)	26.6
**1** (R=none)	2.6
**3** (R=none)	22.0
**6** (R=S, R'=Me)	16.6

c)

hapalindoles and welwitindolinones isolated from *H. welwitschii* IC-52-3 (47g dry tissue)		
Compound	Name	Quantity isolated
**1** (R=none)	12-epi-Hapalindole C isonitrile	11 mg
**1** (R=S)	12-epi-Hapalindole C isothiocyanate	1 mg
**1a** (R=none)	12-epi-Hapalindole E isonitrile	93 mg
**1a** (R=S)	12-epi-Hapalindole E isothiocyanate	4 mg
**2** (R=none)	12-epi-Fischerindole U isonitrile	4 mg
**2** (R=S)	12-epi-Fischerindole U isothiocyanate	2 mg
**2a** (R=none)	12-epi-Fischerindole G isonitrile	4 mg
**3** (R=none)	12-epi-Fischerindole **I** isonitrile	10 mg
**4** (R=none)	welwitindolinone A isonitirle	2 mg
**5** (R=S, R'=H)	welwitindolinone B isothiocyanate	10 mg
**5** (R=S, R'=Me)	N-methylwelwitindolinone B isothiocyanate	5+12 mg
**6** (R=S, R'=H)	welwitindolinone C isothiocyanate	14 mg
**6** (R=none, R'=Me)	N-methylwelwitindolinone C isonitrile	47mg
**6** (R=S, R'=Me)	N-methylwelwitindolinone C isothiocyanate	110mg

Figure S1: Summary of relative and absolute quantities of **1** and its biogenetic derivatives **1a** versus **2** and its biogenetic derivatives **2a**, **3–6** from (b) *H. welwitschii* UTEX B1830 and (c) IC-52-3. The relative quantity shown in (b) was derived from the HPLC analysis of *H. welwitschii* UTEX B1830 crude metabolites and the absolute quantity shown in (c) was derived from what was reported in ref [1]. These data were used to generate the relative molar ratio of each metabolite in the two different producers and the comparison graphs shown in Figure 1b/1c in the main text.

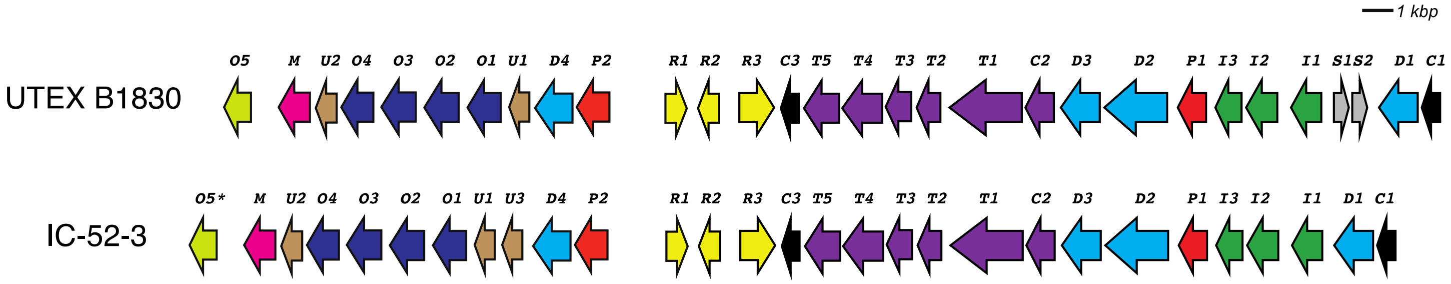

wel Gene	Size (aa)	Protein sequence identity to homolog from *H. welwitschii* UTEX B1830
*wel*O5	290	95%
*wel*M	329	99%
*wel*U2	226	99%
*wel*O4	359	99%
*wel*O3	362	99%
*wel*O2	357	99%
*wel*O1	360	99%
*wel*U1	228	97%
*wel*U3	228	*Unique to IC523*
*wel*D4	400	95%
*wel*P2	334	99%
*wel*R1	234	100%
*wel*R2	243	99%
*wel*R3	368	100%

wel Gene	Size (aa)	Protein sequence identity to homolog from *H. welwitschii* UTEX B1830
*wel*C3	214	100%
*wel*T5	365	99%
*wel*T4	415	99%
*wel*T3	274	99%
*wel*T2	283	99%
*wel*T1	734	99%
*wel*C2	301	99%
*wel*D3	408	99%
*wel*D2	647	99%
*wel*P1	308	100%
*wel*I3	272	100%
*wel*I2	331	99%
*wel*I1	319	99%
*wel*S1	169	*Unique to 1830*
*wel*S2	156	*Unique to 1830*
*wel*D1	406	99%
*wel*C1	183	99%

Figure S2: Sequence identity comparison of proteins encoded in the welwitindolinone BGCs from *H. welwitschii* IC-52-3 and UTEX B1830. The names for some of the genes in the BGC from *H. welwitschii* IC-52-3 (including *wel*U1-3, *wel*O1-5 and *wel*M) (ref [2]) were renamed according to those in the BGC in *H. welwitschii* UTEX B1830 (ref [3]).

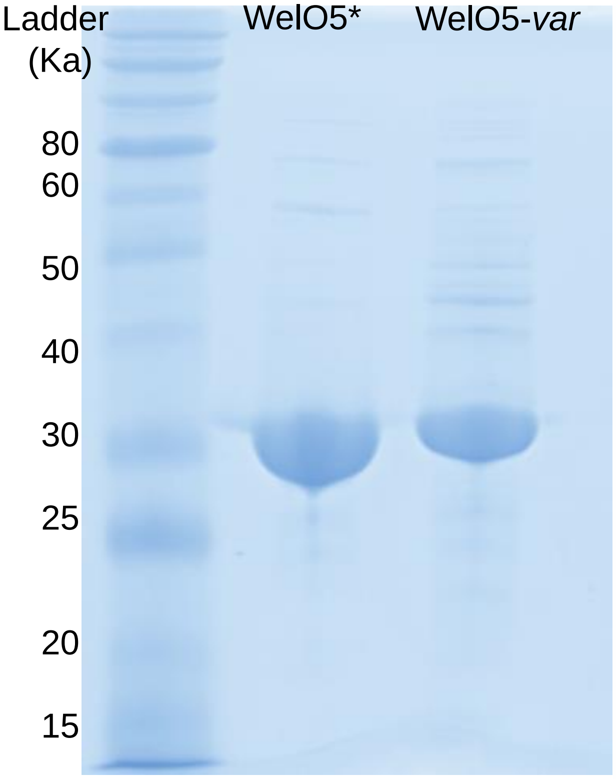

Figure S3: SDS-PAGE of purified NHis7-tagged WelO5* and WelO5-*var.*

References

1. K. Stratmann, R. E. Moore, R. Bonjouklian, J. B. Deeter, G. M. L. Patterson, S. Shaffer, C. D. Smith, T. A. Smitka, *J. Am. Chem. Soc.* **1994**, *116*, 9935-9942.

2. M. L. Micallef, D. Sharma, B. M. Bunn, L. Gerwick, R. Viswanathan, M. C. Moffitt, *BMC Microbiol.* **2014**, *14*, 213.

3. M. L. Hillwig, H. A. Fuhrman, K. Ittiamornkul, T. J. Sevco, D. H. Kwak, X. Liu, *ChemBioChem* **2014**, *15*, 665-669.
